# The Information Gathering Framework – a Cognitive Model of Regressive Eye Movements during Reading

**DOI:** 10.16910/jemr.13.4.4

**Published:** 2020-07-31

**Authors:** Anna Fiona Weiss

**Affiliations:** University of Eichstätt-Ingolstadt, Germany

**Keywords:** Eye movements, regressive saccades, reading, eye tracking, parafoveal processing, attention, models of eye movement control, perceptual span

## Abstract

In this article we present a new eye movement control framework that describes the interaction between fixation durations and regressive saccades during reading: The Information Gathering Framework (IGF).

Based on the FC model proposed by Bicknell and Levy [15], the basic idea of the IGF is that a confidence level for each word is computed while being monitored by three independent thresholds. These thresholds shape eye movement behavior by increasing fixation duration, triggering a regression, or guiding regression target selection. In this way, the IGF does not only account for regressive eye movements but also provides a framework able to model eye movement control during reading across different scenarios. Importantly, within the IGF it is assumed that two different types of regressive eye movements exist which differ with regard to their releases (integrations difficulties vs. missing evidence) but also with regard to their time course.

We tested the predictions of the IGF by re-analyzing an experiment of Weiss et al. [57] and found, inter alia, clear evidence for shorter fixation durations before regressive saccades relative to progressive saccades, with the exception of the last region. This clearly supports the assumptions of the IGF. In addition, we found evidence that there exists a window of about 15–20 characters to the left of the current fixation that plays an important role in target selection, probably indicating the perceptual span during a regressive saccade.

## Introduction

Regressive saccades moving the eyes against the intended reading direction form an integral part of reading behavior. But although they occur frequently during normal reading, with approximately 5–20% of all saccades being regressions [1];, there is little consensus on what exactly triggers such a regressive inter-word eye movement.

At least three different explanations have been brought forward. Regressions may reflect

- a corrective response to overshoots of a former progressive saccade [e.g., 2, 3]
- difficulties or failures in word identification [e.g., 4, 5, 6]
- difficulties in higher-order language processing like syntactic or semantic integration [e.g., 7, 8, 9].

Whereas all of these explanations cover a certain variety of regressions, they all fail to account for the full range of regression patterns reported in the literature. Theories that relate regressions to overshoots of a former saccade, for example, cannot explain why there are so many long-range saccades that move the eyes across several prior words. Theories focusing on difficulties in word identification on the other hand have difficulties to account for the higher number of regressive eye movements in the context of garden path sentences, especially in the disambiguation region.

A problem for all accounts, however, are the findings of a general increase of fixation durations and regressions at the end of a sentence, known as ‘sentence wrap-up effects’ [7, 10, 11, 12];. These regressions occur largely unaffected by sentence processing difficulties, or at least not showing up at the location in the sentence where difficulties are expected to become apparent (although other reading measures indicate difficulties at these locations). Thus, these regressions cannot be directly attributed to failures of the lexical or syntactic integration.

The question what triggers a regression is also closely linked to the question of its function. In reading research, both a higher number of inter-word regressions and increased first-pass reading times (the sum of all fixations made on a region prior to a saccade to another region, also known as ‘gaze duration’ if these regions are single words; [13]; are interpreted to reflect processing difficulties of some kind. However, this raises the important question in which cases the eyes just increase fixation duration and in which cases they trigger a regressive eye movement.

Evidence for functional differences between regression rates and increased first-pass reading times comes from Altmann and colleagues [14]; who reported the counterintuitive finding that gaze durations tend to be shorter when preceding regressions than when preceding progressions, which indicates that these two measures are not just cumulated.

To shed more light on this topic, it is important to consider that eye movements provide a physically different mechanism compared to increased fixation durations because they allow for the intake of additional information (information that often has been processed earlier, at least partly). Against this background, it seems reasonable to assume that regressions are not just an automatic response without any linguistic control but that they form an integral part of problem solution.

Thus, as a first step we propose the working hypothesis that difficulties in language processing take the form of increased fixation durations (gaze durations) if the problem can be solved with the currently available information, and the form of higher inter-word regression rates if the problem cannot be solved with the currently available information.

### Regressions in the context of current models of eye movement control

In the last decades an impressing number of computational reading models have been developed which succeed in predicting and simulating human reading behavior. After first primarily focusing on low-level factors like frequency or word length and their interaction with eye movement behavior during reading, recent models of eye movement control were extended in order to capture higher-order language processing as well. This also includes regressive eye movements. In the following, we will briefly discuss three influential models, the E-Z Reader 10, SWIFT and Glenmore model, with regard to regressive eye movements. After that we will discuss the, to our knowledge, only model that explicitly focuses on regressive eye movements during reading, the model of Bicknell and Levy [15];.

#### E-Z Reader 10

The E-Z Reader model [16, 17]; was first developed to account for the interplay between lexical processing, attention allocation, and saccadic programming during reading and made no predictions about higher-level language processing. However, the latest version of the model, E-Z Reader 10 [18];, now also tries to explain the interaction between ‘post-lexical processing’ and eye movement control.

For this reason, a post-lexical integration step has been added to the model’s architecture. During this step, the currently processed word (word n) is integrated into higher-level representations such as the syntactic structure or the discourse model. In case this integration fails, it causes both an attention shift and a regressive eye movement “back to the point at which the difficulty became evident (i.e., word n), as opposed to some earlier sentence location” (18, p. 6).

This post-lexical integration step provides a substantial modification of the former model and clearly extends the model’s explanatory power. However, the model can only account for regressions targeting word n and, in addition, only for post-lexical integration difficulties, which is just an approximation to the complexity of regressive eye movements during reading. On the one hand, regression target locations show a more complex distribution pattern (see e.g. [1] for a review) and on the other hand, post-lexical integration difficulties cannot account for all types of regressions (see e.g. the function of ‘small regressions’ proposed by [1] or the so-called ‘sentence wrap-up effects’ mentioned earlier). But we have to keep in mind that the authors of the model explicitly state that “the integration stage […] is a placeholder for a deeper theory of postlexical language processing during reading. Our goal in including this stage is therefore quite modest: to provide a tentative account of how […] postlexical variables might affect readers’ eye movements.” (p. 6). In other words, the E-Z Reader 10 model is not designed to simulate the whole range of regressive eye movements during reading but provides a limited but helpful tool in modeling eye movements during higher-order language processing.

#### SWIFT

The SWIFT model, proposed by Engbert and colleagues [19, 6];, is another highly advanced model of eye movement control. It assumes that multiple words are processed in parallel while saccades are generated autonomously, selecting the target in a probabilistic manner according to the activation levels of words (Luce’s choice rule). Importantly, the activation levels of potential saccade targets on a saliency map (the activation field) are influenced by the current attractiveness, the informativeness, or saliency of these potential targets.

The fluctuation of these activation values is the driving principle for all types of saccades which also includes regressive eye movements. This means that regressive eye movements are assumed to be triggered by incomplete word recognition (at least for the time the word is in the perceptual span). In this case, the eyes are re-directed to the word with non-zero activation. Once the identification process is completed, the word forms no longer part of the group of potential saccade targets.

As the E-Z Reader model, the SWIFT model does not claim to account for the full pattern of regressive eye movements during reading. Thus, we see again some limitations of the model with regard to regressions (and to word processing in general).

As for the E-Z Reader model we find that the SWIFT model covers primary regressions which target the immediately preceding word. Although words earlier in the sentence (especially those which are short and have a very high frequency) can also be the target of a regression due to residual activation, the SWIFT model does not have any mechanism to account for regressions due to higher-order comprehension failures.

In addition, the SWIFT model proposes that word recognition cannot fail because all words will be recognized as long as activation is left. From a psycholinguistic perspective this assumption might be problematic since there is large evidence that a misinterpretation of words is fairly common in sentence / text reading and leads to increased reading times and a higher number of regressive eye movement (c.f. for example the misinterpretation of ambiguous phrases in garden path sentences).

#### Glenmore

The Glenmore model [20]; also belongs to the class of models allowing for parallel processing of several words. Although it shares many similarities with the SWIFT model, it differs in one important respect: Whereas the SWIFT model assumes that word-activation levels are translated into probabilities of words being selected as saccade targets according to Luce’s choice rule, the Glenmore model proposes a ‘winner-takes-it-all’ policy. This is, the next saccade is always programmed towards the word with the highest saliency on the saliency map. This saliency level is computed on the basis of combined visual and linguistic (letter and word-level) processing.

The Glenmore model explicitly describes a scenario for the triggering of regressions. Regressions are performed whenever the word left to the currently fixated words wins the competition of saliency with other potential saccade targets. This happens, for example, if word n-1 has not been fixated earlier and word n is a long but highly frequent word. In this case, the saliency of word n-1 is high (because it has not been fixated earlier) and the saliency of word n is low because word recognition is facilitated by the high frequency. As a response, a regression to word n-1 is performed.

As the former models, the Glenmore model is impressively elaborated on several aspects and is able to capture many important findings with regard to eye movements during reading. Especially, it provides a parsimonious mechanism for the triggering of saccades, including regressions. But as the E-Z Reader model, Glenmore can only cover regressions to word n-1. In addition, the authors explicitly state that “the focus of current version of the model is saccade target selection. The development of a more realistic word recognition module is planned as a future extension” (p.35). Thus, the assumed linguistic processes (word recognition on the basis of appropriate connections between letters and words) work well in the current model architecture but are not able to cover full, especially higher-order linguistic processing.

#### Falling confidence

Due to the limitations of the E-Z Reader and the SWIFT model with regard to regressive eye movements, Bicknell and Levy proposed another model of eye movement control that aims to overcome the weaknesses of the former models [15];. We will refer to this model as the ‘model of falling confidence’ or ‘FC model’ for short. At the core, it is assumed that the word identification process is never completed. Thus, “it is possible that later parts of a sentence can cause a reader’s confidence in the identity of the previous regions to fall” (15, p. 1170) which triggers a regressive eye movement in order to get more visual information about the previous region.

According to the framework, the model generates distributions over possible identities of the sentence, based on its language model. During a fixation, the noisy visual input is used to update the model’s beliefs by a Bayesian likelihood term and by the language model. Thereupon, the model selects an action which could either be to continue fixating, to trigger a saccade or to stop reading the sentence before the cycle repeats.

A simple control policy is assumed to decide between actions, which works on the basis of two thresholds: The first value defines the threshold for a character to remain fixated. The second value defines the threshold for an (already processed) character on a leftward position to be fixated again (by a regression). Thus, the model allows to independently modulate the control policy with regards to processing depths (i.e., increased fixation durations) and regression probability determining the speed and accuracy of the model. It is hypothesized that a strategy without making regressions is slower and less accurate than a strategy with shorter fixation durations and occasionally making regressions.

The model of Bicknell and Levy fits well with the working hypothesis proposed at the beginning of this paper and offers a clear mathematical description of how such an account may be integrated into a simulation model. Furthermore, it builds on the basic ideas of the SWIFT and Glenmore model but replaces their concept of “incomplete word recognition” (SWIFT – at least for words within the perceptual span) or ongoing salience (Glenmore) by the idea that word identification never is completed. Although the notion of (in)complete word recognition or ongoing salience is perfectly fine in its own context, seen more generally it is problematic to view word recognition as an ‘all or nothing’ task, given the large amount of information that is connected to a word (e.g. its meaning, semantic neighborhood, word class as well as predictions about other entities in the sentence and so forth). Rather, it is more convincing (especially from a psycholinguistic perspective) to assume that word recognition is a process that needs time and can never be completed. Also and in clear contrast to the SWIFT model, the FC model accounts for the fact that word recognition may fail. Thus, this new assumption of the FC model seems to be a more realistic notion.

In a first step, Bicknell and Levy took the model to simulate regression behavior on English sentences by comparing the efficiency of different reading strategies. For this, they adjusted the thresholds for the control policy and measured the resulting reading speed and accuracy in different simulations, showing that (as predicted) a strategy which occasionally allows for regressions leads to a higher reading speed and better accuracy than a strategy without making regressions. In a second step, Bicknell and Levy [21]; tested predictions of the FC model by analyzing the Dundee corpus [22]; and showed that the FC model was the only theory that was able to account for the observed pattern. We will discuss this work in more detail in the section ‘Applying the Information Gathering Framework to the findings in the literature and deriving further predictions’.

Although the FC model provides a very helpful account in modeling between-word regressions, it is a simplification in many regards as well. We will discuss these limitations in more details below when we introduce the architecture of the new model.

## A new approach: The Information Gathering Framework

After having reviewed how current models of eye movement control try to capture regressive eye movements in reading, it becomes apparent that all of them add helpful ideas to our understanding of mechanisms that control regressive eye movements during reading but that they all have limitations with regard to several aspects as well.

In the following, we will therefore propose a new framework that may provide a general tool for our understanding of regressive eye movements, without limiting it to a small range of linguistic phenomena. As a starting point, we will use the FC model proposed by Bicknell and Levy [15];. But instead of focusing on theoretical considerations about reading strategies, the current aim is to develop a realistic model of human reading behavior, which means that the model should be able to cover findings from the existing literature as well as to make further testable predictions about reading behavior. This, however, requires some substantial modifications in the architecture of the FC model, so that we will call the new account the Information Gathering Framework (IGF).

We acknowledge that our approach has limitations in several ways as well and we want to encourage others to also test and modify this framework. Also note that in contrast to the FC model, the IGF is not incorporated into a computational model as yet that allows for simulating reading. Instead, the IGF takes into account more cognitive and linguistic properties of eye movement control than the former model does. But the current considerations should be used by future research to combine these two approaches and to develop a computational version of the IGF as well.

### The architecture of the Information Gathering Framework

Before explaining the assumptions of the IGF in more detail and clarifying its modifications from the FC model, we will briefly summarize the architecture of the IGF by the following six assumptions:

1. The confidence in each word’s identity is described by the confidence level. The confidence level is computed by matching predictions about incoming material with the lexical representations of a word.
2. The lexical representation of a word is viewed as an infinite bundle of features which takes time to be retrieved and which varies among individuals (indicated by the lexical quality level).
3. The focus of attention (i.e., the area within the confidence levels are computed in parallel) is restricted to two words.
4. There are three different thresholds for the confidence level causing an action: The forward threshold defines the confidence level that is needed to trigger a progressive eye movement, whereas the backward threshold prevents a regression. The re-inspection threshold prevents the word from being selected as a regression target on the basis of explicit linguistic processing.
5. There are two different scenarios that cause a regressive eye movement: First, if the confidence level falls under the forward threshold after the eyes have already moved to the next word, and second, if the backward threshold is not reached before the confidence level of the next word reaches the forward threshold.
6. There are also two different scenarios as to how a regression target is selected: Either by targeting the word within the perceptual span with the confidence level under the re-inspection threshold or by using experience-based strategies.

Please notice that although all regressions share the same characteristics (e.g., an eye movement against the intended reading direction, re-reading of former sentence material etc.), the idea to summarize all regressions under one unifying function is probably not convincing. Inhoff et al. [1];, for example, suggested that two different types of regressions can be distinguished, namely according to their size, function and target control. One type is referred to as ‘large regressions’ and comprises regressions “that traverse across more than one prior word” (p. 36). They argue that these regressions are highly coupled to linguistic processing and serve to improve comprehension by re-processing prior text. The second type of regressions are ‘small regressions’, typically including refixations of the current word and inter-word regressions to the immediately preceding word. These regressions are assumed to reflect responses to inaccurate or premature oculomotor programming and serve to improve visual word recognition.

We agree with this distinction and in the following we will focus on ‘long regressions’ only. However, although some regressions to word n-1 certainly share the characteristics of ‘small regressions’, we doubt that all of these regressions can be attributed to this class. Thus, we use a slightly broader definition and just exclude regressions due to errors in oculomotor programming, but include regressions to word n-1 not falling in this category.

For all these inter-word regressions we propose one unifying function, this is to gather additional information relevant in the course of sentence interpretation, more precisely, to gather additional information about the identity of words.

#### (1) The lexical quality level

The FC model proposes that because word identification is based on noisy visual information, “word recognition may be best thought of as a process that never is completed” (15, p. 1170). Although we agree on the assumption of incomplete word recognition, we doubt that noisy visual information is in fact the major determinant of word identification, especially because there exists convincing evidence that the decoding of visual information occurs very rapidly (e.g., 23). Thus, we rather claim that word identification is mainly affected by the retrieval of the lexical information (as also proposed by the SWIFT and E-Z Reader model).

To incorporate this idea in our framework, we assume that the underlying language model contains lexical representations of each word. Specifically, the lexical representations stored in memory have to be viewed as (theoretically) infinite bundles of features, containing information about the word’s orthography, phonology, meaning, morpho-syntax as well as its constituent binding preferences (c.f. also [24] who introduced this idea as the concept of lexical quality in order to explain differences in language skill between individuals). Because of the complexity of the lexical representation it takes time to retrieve this information from the lexicon.

**Figure 1:**
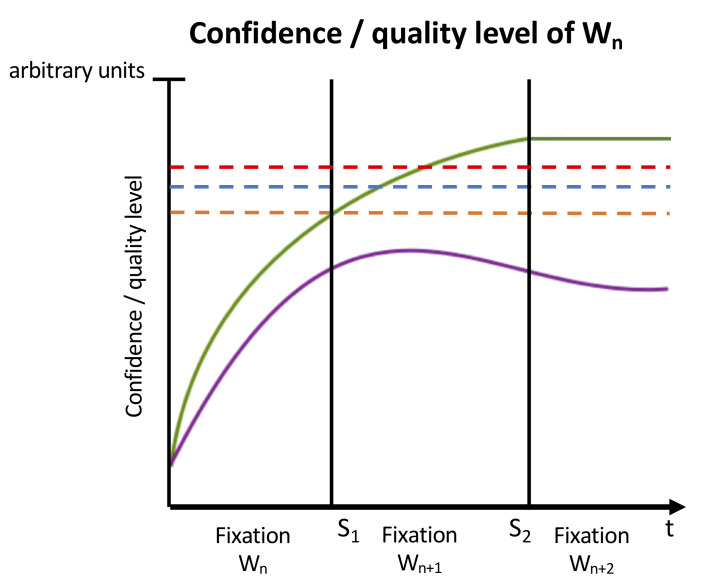
Schematic illustration of the confidence / quality level of a single word during a typical sequence of two progressive saccades: Whereas the confidence level is continuously increasing and asymptotically approaching the full confidence level, the quality level decreases due to interference and decay after the eyes moved to the next word. Legend: green = confidence level, purple = lexical quality level, orange = forward threshold, blue = backward threshold, red = re-inspection threshold, S_1_ = saccade to word n+1, S_2_ = saccade to word n+2, t = time.

We refer to the amount of information about a word that is currently retrieved from the lexicon with the term ‘lexical quality level’. Typically, the amount of information (and thus the lexical quality level) continuously in-creases during a fixation because a fixation allows for the retrieval of lexical information on the basis of the visual input. However, once the eyes have moved to the next word, no additional information can be received and the quality level is then continuously decreasing over time due to interference from other words and due to a decay of the memory trace (25; see Figure 1 for a schematic illustration). Also note that the lexical quality level of a word (as the confidence level, see below) is never reaching the full quality level because the retrieval of the information from the lexical entry can by definition never be completed.

#### (2) The confidence level

In addition to the lexical quality level, the IFG claims that a confidence level for each word is computed which basically represents the reader’s confidence in the identity of the current word.

According to the FC model, the reader computes a confidence level of a particular word on the basis of its language model. If additional information causes the confidence in a previous word’s identity to fall under a certain threshold, a regressive saccade to this particular word is triggered. Because the FC model computes the confidence level on the basis of the underlying bigram frequency model, its focus is set on reducing noisy visual input and the computation of confidence levels mirrors just a coarse approximation to the complexity of word recognition processes.

Since we want to take a broader perspective here which also covers higher-order language processing, we propose within the IFG that the computation of confidence levels (as the computation of the lexical quality levels) is based on linguistic processing and takes a certain amount of time. During this time, the confidence level of a word typically increases (asymptotically approaching but never reaching the full confidence level), because more supporting evidence is given from the information of the lexical representation (see Figure 1 for a schematic illustration). For current purposes, it is assumed that the confidence level is computed by matching the features of the lexical representation with the predictions of former sentence material on the basis of explicit production rules [26];.

These production rules represent all procedural knowledge (grammatical knowledge) and define condition–action pairs. For example, if an inanimate noun (e.g. *the table*) is encountered as the initial argument in an English sentence (condition), the production rules predict that a verb (action) will follow in the course of the sentence. More precisely, they predict that this verb should agree with the argument in number (singular), comes with an inanimate subject, and so on. If a verb like *talks* is encountered next, this leads to a violation of production rules because *talks* requires an animate subject. On the other hand, if a pronoun like the word *which* is following, it induces a relative clause. In this case, the production rules are not violated and the action (the expected verb) is simply postponed. Also, not every condition-action pair is mandatory; some pairs are just optional (e.g., the indirect object of verbs like write: *He writes a letter (to his father)*). If the evidence provided by the lexical representation matches the predictions made on the basis of the production rules, a high confidence level is computed. If the production rules are violated by contrast, it leads to a low confidence level. Accordingly, if the context is highly predictive, less lexical information and thus less time is needed to reach a certain level of confidence resulting in shorter fixation durations.

Note that the level of confidence is highly correlated to the lexical quality level, but these two parameters are not the same. A poor reader could have a high confidence in a word’s identity although it is ambiguous (e.g., in meaning). But due to a small lexicon which implies a representation of a few features only, the reader is not aware of these alternative interpretations. Accordingly, a proficient reader could have low confidence in the same word’s identity because he takes into account several potential ambiguities that the poor reader is not aware of. In addition, a highly predictive context may also cause that less information (and thus a lower lexical quality level) is needed to confirm this prediction and a certain level of confidence is reached. This explains why gaze durations on highly predictive words are shorter than those on unpredictable words (e.g., 27).

Although the notion of explicit production rules is not experimentally verified yet, there exists comprehensive evidence from a variety of behavioral tasks (including reading) that prediction on several linguistic levels forms an integral part of language processing (for a recent overview, see 28). In addition, there are also influential accounts that highlight the strong relationship of language production and comprehension, assuming that both modalities share fundamental mechanisms [29];. Thus, the concept of production rules guiding predictions about the following input may provide a useful tool to model language processing in terms of prediction although it needs more experimental support.

Also, the claim that a mismatch of predictions is the main determinant of regressions is not without problems. In particular, it would imply that regressions serve to improve comprehension because they provide additional information that helps to solve prediction conflicts (note that we have to assume that there are indeed solutions in coherent sentences and texts). However, the empirical evidence for this claim is somewhat inconsistent.

Schotter and colleagues [30]; examined the question whether regressions help in comprehension in a clever masking experiment with garden path sentences: All words to the left of the current fixation were replaced with an x-mask so that possible regressions did not provide any useful information. The authors found that although the opportunity to regress supported comprehension, actually making a regression did not lead to significantly better comprehension results compared to cases where the reader did not regress.

More recently, Metzner, von der Malsburg, Vasishth, and Rösler [31]; compared sentence comprehension of free-reading and word-by-word presentation in a concurrent ERP / eye-tracking study. They found that accuracy improved when reading naturally compared to the word-by-word presentation, but that the benefit was only visible when the eyes actually made a regressive saccade.

It is not fully clear where these differences come from. The mode of presentation might have had an effect on the results. But also the difficulty of sentence material seems likely to have affected the benefit of a regression: The overall accuracy results indicate that the stimulus material used by Schotter et al. was much harder to process than the sentences used by Metzner et al. Thus, the claim that regressions support comprehension seems to be dependent on the language proficiency of the reader. In other words: Even a regressive eye movement would be useless if the reader does not have the ability to deal with the linguistic problem. This may also explain the lack of a comprehension benefit in the case of a regression in the data reported by Christianson, Luke, Hussey, and Wochna [32, experiment (1); among many others.

#### (3) The confidence level is monitored by three independent control mechanisms

The FC model proposes that the generation of eye movements is monitored by a simple control policy that sets two different values of confidence causing an action. If the first value is reached, a forward saccade to the next word of low confidence is initiated. If the confidence level of a word falls under the second value, a regressive eye movement to this particular word is triggered.

In the IFG the actions are controlled by three independent thresholds for the confidence level, which we refer to as the forward, backward and re-inspection threshold (see Figure 1).

The first (forward) mechanism defines the level of first-pass confidence, namely the amount of evidence about word n’s identity that is retrieved in first-pass reading and assessed to be sufficient for the current sentence interpretation. When a certain level of confidence is reached, the eyes move to the next word.

It is further proposed that this forward control mechanism works in a highly automatic manner, per default targeting the next word. This automatic saccade generation is canceled and the eyes move to word n+2, if parafoveal processing already reveals a certain level of confidence for word n+1. The forward control mechanism proposed here is compatible with current models of saccade control like SWIFT [19, 6]; that assume a) parallel processing of different words, b) largely automatic generation of progressive (and regressive) eye movements, and c) word identification as the core function of saccades in reading.

This forward threshold in particular mediates between speed and accuracy: If the threshold is set down, the reading speed is increased but accuracy also suffers. If the threshold is set high, by contrast, the accuracy is higher but at the expense of reduced reading speed.

The second (backward) mechanism defines the level of confidence that has to be reached in order to prevent a regressive eye movement from happening. Thus, a regression is performed whenever the level of confidence for a word does not reach a certain threshold. In contrast to the forward control mechanism, this backward mechanism is highly linguistically controlled.

Although the forward and backward control mechanisms often interact, they are assumed to be independent and may be adjusted separately. Thus, there may exist a first-pass strategy that allows for relatively superficial reading, but this does not necessarily mean that at the same time the probability for regressions increases. In addition, both control mechanisms are assumed to be sensitive to top-down influences (i.e. tasks) that may reduce or increase the thresholds for first-pass reading times and regressions. Bicknell and Levy [15]; for example showed that the most efficient reading strategy (i.e., the one that leads to highest comprehension accuracy) is one that allows for a lower level of confidence in first pass and increases the probability for regressions at the same time.

The third (re-inspection) mechanism defines the level of confidence that prevents a word from being selected as a regression target on the basis of explicit linguistic processing. Thus, if the confidence level of a word does not reach this re-inspection threshold during a fixation of the subsequent word, it provides a potential target for the regression (we will explain this procedure in more detail below).

#### (4) Limited focus of attention

The FC model takes into account the limitations of the visual field in order to compute the degree of noisiness for the visual input, but it is not specified with regard to the focus of attention. However, because the underlying language model is restricted to bigram frequencies, the confidence level of a word can only be affected by the visual information about the subsequent word.

**Figure 2:**
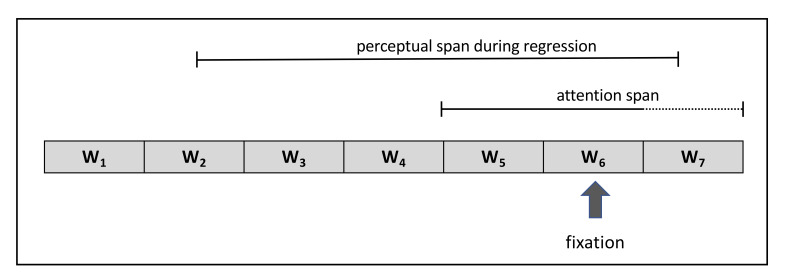
Schematic illustration of the attention and perceptual span. The confidence level can be only computed for words within the attention span. In the case of a regression, the word within the perceptual span (W2, W3, W4, or W5 in the example above, given 5-letter words and assuming a size of the perceptual span of about 15–20 characters) whose confidence level did not reach the re-inspection threshold is selected as the regression target. If there is none or more than one word (except W5) whose confidence level did not reach the re-inspection threshold, the target is selected on the basis of a strategy (see text for further information).

Within the IGF, the visual field also shapes the amount of visual information that is available to the reader during a fixation and that is used for the computation of the lexical quality level. But in addition, it is assumed that the computation of confidence levels always requires attention, so that not the confidence levels of all words in a sentence can be monitored in parallel. In particular, research on the basis of SAT (speed accuracy trade-off) experiments has indicated that the focus of attention is very limited, covering only two chunks [33];. We therefore assume within the IGF that the focus of attention is restricted to the word of the current fixation (W_6_ in the example below) and the word before (W_5_ in the example below) which means that only the lexical representations of these two words can be used in parallel to compute the confidence levels (see Figure 2).

Note that the concurrent allocation of attention to word n and word n-1 is a highly controversial claim and stands in clear contrast to models like E-Z Reader. However, there is evidence that this kind of attention allocation is indeed possible (e.g., 34).

#### (5) Four different eye movement scenarios

**Figure 3:**
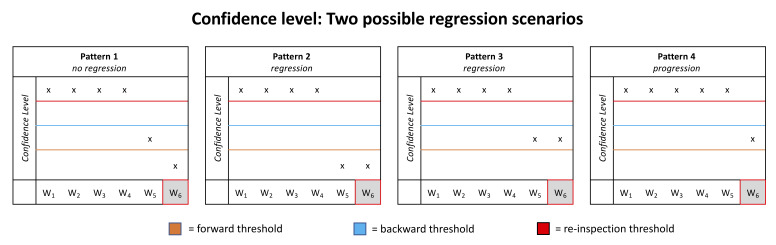
Potential patterns of confidence levels. Each pattern represents the confidence levels of six words (W_1_ to W_6_) during a fixation on W_6_. Please note that only the confidence levels of two words (W_5_ and W_6_ in the example above) can be computed in parallel. Also, in this example all confidence levels reached the re-inspection threshold which is not necessarily the case. But this has no further implication for triggering a regressive or progressive eye movement but only for target selection (see text for further information).

In a framework with an architecture described above, four different eye movement scenarios are possible (see Figure 3). We will now describe them in turn. Note that each graph represents the confidence level of six words (W_1_–W_6_) while the eyes are currently fixating word 6 (W_6_).

##### Pattern 1

The confidence level of W_5_ has already passed the forward threshold which triggered a saccade to W_6_. Now the confidence level of W_6_ is also increasing, and the word remains fixated until the confidence level of W_6_ reaches the forward threshold. Alternatively, the confidence level of W_5_ drops under the forward threshold.

##### Pattern 2

The confidence level of W_5_ drops under the forward threshold after first passing it (which triggered the saccade to W_6_). This may happen because the computation of the confidence level for W_5_ still continues after the eyes moved to W_6_. Sometimes the computation of the confidence levels reveals that W_5_ cannot be integrated into the current sentence structure which causes that the confidence level of W_5_ drops under the forward threshold. As a response, a regressive eye movement is triggered.

##### Pattern 3

There is another scenario that causes a regression: If the confidence level of W_6_ already passed the forward threshold but the confidence level of W_5_ did not reach the backward threshold. This happens for example if the new input does not provide the expected evidence about W_5_’s identity. In this case, the confidence level of W_5_ increases only slowly. However, if the confidence level of W_6_ reaches the forward threshold in the meanwhile, a regression is triggered. We assume that this happens especially at the end of a sentence where the whole sentence structure is evaluated.

##### Pattern 4

In this case, the confidence level of W_6_ reached the forward threshold after the confidence level of W_5_ reached the backward threshold. This is assumed to be the “normal” case and it triggers an eye movement to W_7_.

#### (6) How the target of a regressive eye movement is selected

The IGF predicts that there are two different regression scenarios: regressions due to integration difficulties (pattern 2) on the one hand and regressions due to missing evidence on the other (pattern 3). However, a crucial question is how the target of this regressive eye movement is selected.

The FC model predicts that the regression always targets the word with the confidence level under the backward threshold which is always the directly preceding word (due to the underlying bigram frequency model). However, the assumption that regressions are always targeting word n-1 (an assumption which is also shared by the E-Z Reader 10 model, for example) is just a simplified approximation, as discussed above. We also have to keep in mind that the word in the sentence where problems become apparent does not always correspond to the word that causes difficulties. A very prominent example are garden path sentences where difficulties are often caused by a misinterpretation of a word earlier in the sentence. In this case, a re-inspection of the word n-1 would not help to solve the problem, and since we assume that the function of a regression is to solve the problem, this is not a plausible mechanism.

Another opportunity would be to select the word with the lowest quality level as the target for the regression instead because there is an increased likelihood that more evidence (provided by the lexical representation) about this word would help to increase confidence. However, there are also difficulties with this assumption: As already discussed, the quality level and the confidence level are not the same. Thus, a low quality level does not automatically cause a low confidence level. In addition, this assumption would lead to the conclusion that words earlier in the sentence / text are more likely to become the target of a regression because the quality level is low (due to the decrease over time). This prediction, however, is not supported by the empirical findings either.

A third opportunity would be that a re-computation of confidence levels of all prior words takes place and that the word with the lowest confidence level (or the confidence level under the backward threshold) is selected as the regression target. However, since the computation of confidence levels requires attention and there is only a very limited focus of attention (see above), this is not possible within the model’s architecture, either.

For this reason, a third threshold is assumed within the IGF: the re-inspection threshold. Typically, the confidence level of word n-1 reaches the backward and the re-inspection threshold during a fixation on word n (see Figure 3). But in some cases, the linguistic processing reveals still a substantial doubt in the confidence of a word although it provides a possible but unexpected input for the current sentence interpretation. As a consequence, the confidence level of this particular word reaches the forward and the backward threshold but not the re-inspection threshold. But this does not have any effect on eye movement behavior at this point of time.

If, however, a regressive eye movement is triggered in the course of sentence reading, the word whose confidence level did not reach the re-inspection threshold is selected as the regression target because more confidence is needed here.

Since this procedure would require to monitor all confidence levels of a sentence or even a text in parallel, there has to be some limitation of the amount of words which can be selected by such a mechanism. For the current framework we claim that this target selection mechanism is restricted to words within the perceptual span.

Several studies have shown that the perceptual span comprises 3 to 4 letter spaces to the left of the fixation [35, 36]; and 14 to 15 letter spaces to the right of the fixation during reading [37, 38];. Because the perceptual span is not a restriction of the visual system per se, but is rather affected by attentional processes (for example indicated by the finding that systematically increasing the font size of the letters to the right or left of the fixation does not reduce the perceptual span: 39), it has been hypothesized that the perceptual span changes when making a regressive eye movement. This hypothesis has been confirmed by research of Apel and colleagues [40];, who showed that the size of the perceptual span switches toward the direction of the eye movement which also implies a shift of attention to the left. Although the authors did not answer the question of the actual size of the perceptual span to the left of a fixation during regressions we suggest that the perceptual span encompasses about 15–20 characters to the left, according to the size of the right perceptual span in progressive eye movements. However, the precise size of the perceptual span has to be further examined by future research.

It follows for the architecture of the IGF, that when making a regression, the word within about 15–20 characters to the left of a regression is selected as the regression target if its confidence level did not reach the re-inspection threshold.

However, because word n-1 never reached the re-inspection threshold when a regression is triggered (see Figure 3), this would lead to the prediction that regressions are always targeting word n-1 (which is obviously not the case as discussed earlier). But note that word n-1 is still in the focus of attention which allows for the computing of its confidence level but also for the retrieval of its lexical information. Thus, it is assumed that word n-1 is only selected as the regression target if the linguistic processing reveals that information about the identity of word n-1 would help to solve the problem. In all other cases the word prior in the perceptual span whose confidence level did not reach the re-inspection threshold is selected as the regression target.

In the case the confidence of none or more than one word (apart from word n-1) did not reach the re-inspection threshold, the regression target is selected by the backward control mechanism on the basis of experience-based strategies which also means that the target selection is not restricted to words within the perceptual span. It seems likely that a target selection based on strategy is more the rule than an exception.

The limited set of selection strategies is based on language experience and aims to define the most efficient way to gather the required information, without taking into account the details of the lexical representation or requiring language processing itself. Most efficient is defined as the combination of speed and accuracy, which means that the strategy is the fastest way to find the most relevant information in the absence of explicit knowledge, taking into account the speed-accuracy tradeoff. Language experience means that this strategy has been applied most frequently in the past and yielded good results, so that the reader when he is faced with a certain category of tasks, assesses the likelihood where the relevant information can be found on the basis of his language experience. Strategy means that the same type of eye movement (B) is performed when faced with the same task (A) – at least for a single reader – resulting in the simple condition term: if A, then B.

Note that it is probably not a certain sentence type which induces a certain backward strategy, but that these strategies mainly differ between individuals due to memory capacities or reading skill. Thus, many studies found evidence that readers prefer a certain strategy. Poor readers, for example, seem to use the backtracking strategy more often than good readers do (41; see also e.g. [11, 12] for identifying scanpath signatures among individuals).

The assumption that the target selection of regressions is under linguistic control (which is assumed in the case of targets that are selected because their confidence level did not reach the re-inspection threshold) is a contentious issue. Mitchell and colleagues [42];, for example, introduced the idea that regressive eye movements just may reflect some kind of cognitive-inhibition mechanism. This ‘time out hypothesis’ assumes that “the function of the system is nothing more than that of postponing new input” (p. 269) which also implies that there is no linguistic guidance on regression target selection. However, the authors were not able to provide any evidence for this hypothesis because their syntactic manipulation had a clear impact on the landing sites of regressive eye movements.

But the opposite claim also failed to receive sufficient support. Frazier and Rayner [7]; proposed the ‘selective reanalysis hypothesis’ which assumes that in the case of garden path sentences the parser regresses to a position where he expects the source of the error. Although Frazier and Rayner found that 53% of regressions initiated in the disambiguating region and beyond ended in the ambiguous region, the regressions nonetheless showed a relatively high variance with regard to their landing sites, questioning such a strong linguistic guidance. Because the number of regressions was very small and statistical evidence was missing, Meseguer and colleagues [10]; conducted a follow-up study two decades later. But they were not able to find convincing evidence for this strong linguistic guidance, either.

Thus, we think that more factors may shape the landing site distribution, although linguistic computations are assumed to be the main determinant. These factors are differences between individuals with respect to linguistic knowledge (e.g., 1, 43) or memory capacities [44, 45];. But also general factors like spatial memory (46, 47 as well as [48] for an overview), oculomotor error [49]; and visual salience (e.g., 50) may play an important role in determining landing site distributions of regressions. This, of course, makes it hard to draw strong predictions from the model’s architecture and we acknowledge that more research has to be done in this domain.

### Applying the Information Gathering Framework to the findings in the literature and deriving further predictions

Having described the main properties of the IGF, we will now discuss how the model may account for a variety of critical empirical findings reported in the context of regressive eye movements during reading.

In addition, another important factor supporting the strength of a model is that it allows for further predictions. In the following, we will therefore also discuss several predictions that can be derived from the architecture of the model. But note that not all predictions discussed here will potentially verify or falsify the model. For example, the IGF assumes that new input is matched against predictions arising from previous input, which is one of the core principles of the model. If we were to find empirical evidence against this assumption, this would question the validity of the model. But whether these predictions are accomplished on the basis of production rules, by contrast, does primarily affect the detailed architecture of the model but not its core principles.

#### (1) Properties of word n and word n-1

Above we mentioned the work by Bicknell and Levy [21]; testing predictions of the FC model. In their study they were focusing on the relationship between inter-word regressions and properties of word n and word n-1. They discuss the predictions of several theories that account for regressive eye movements during reading. Table 1 provides an overview over these predictions according to Bicknell and Levy.

**Table 1:** Predictions of different theories with regard to the properties of word n and n-1and their interaction with inter-word regressions according to Bicknell and Levy (21) and the IGF as well as results of the corpus analysis. Abbreviations: ↑ = increased regression probability, → = no effect on regression probability, ↓ = reduced regression probability, empty cells = no clear prediction or statement, + length = longer word length, - freq = less frequent, - pred = less predictable.

**Model / class of theories**	**Properties of word n-1**			**Properties of word n**		
**Predictions according to Bicknell and Levy (21)**	+ length	- freq	- pred	+ length	- freq	- pred
Incomplete lexical processing – serial (e.g., E-Z Reader)	↑	↑	↑	→	→	→
Incomplete lexical processing – parallel (e.g., SWIFT)	↑	↑	↑	↓	↓	↓
Integration Failure	→		↑	→		↑
Falling Confidence	↑		↑	→		↑
IGF – regressions type I	↑	↑	↑	→	→	↑
IGF – regressions type II	↑	↑	↑	↓	↓	↓
**Results of Bicknell and Levy (21)**						
Not corrected for correlation	↑	↑	↑	→	→	↑
Corrected for correlation		↓	↑			

Predictions were tested by using the Dundee corpus [22];. In contrast to former studies [51, 5]; the authors controlled for skipping of word n-1 and clearly distinguished between the factors word length, frequency and predictability. The analysis revealed that there were more regressions when word n-1 was longer, more frequent and less predictable as well as when word n was less predictable (see Table 1). Length or frequency of word n did not have an effect.

However, because there was a high correlation between the factors frequency and predictability for word n-1, the authors carried out an additional analysis which accounted for this correlation. This analysis revealed that there were highly significant effects of the predictability and frequency of word n-1, but in opposite directions (i.e., increased regressions for less predictable but more frequent words).

Bicknell and Levy argue that these results fit best with the assumptions of the FC model. In general, the FC model proposes that an unpredictable word n is more likely to cause confidence to fall which triggers a regressive eye movement. In addition, because for longer, less frequent and less predictable words the confidence level is lower to begin with, it is more likely that the confidence level of these words fall. This may explain the general higher regression probability for word n-1 when it is longer, less frequent and less predictable. The opposing effects of predictability and frequency, however, are interpreted in the sense that unpredictable words only cause more regressions if they are more predictable for alternate possible contexts (indicated by a high frequency). Thus, Bicknell and Levy conclude that their data suggests “that the amount by which a word makes confidence to fall is a key determinant in whether a reader will make a regressive saccade.” (p. 936)

The IGF shares the predictions of the FC model with regard to the properties of word n and n-1. If the confidence level of word n-1increases slower (due to low frequency or less predictability), then it is more likely that the confidence level drops under the forward threshold during a fixation on word n (regressions of type I) or does not reach the backward threshold (regressions of type II). But the IGF provides a clear theoretical explanation for the opposing effects of frequency and predictability: Because the lexical quality level and the confidence level are assumed to be (in principle) independent, properties like frequency (which is associated with the lexical quality level) and predictability (which is associated with the confidence level) may affect the regression behavior in different ways.

The IGF also predicts (as the FC model) that a less predictable word n also increases regression probability because it fits poorly with the prior context. As a response, the confidence level of word n-1 drops under the forward threshold and a regression is triggered (regressions of type I). This should happen widely unaffected by the length or frequency of word n (or at least not resulting in a clear pattern). However, the IGF makes an additional prediction: If the confidence level of word n needs more time to cross the forward threshold, then the confidence level of word n-1 has more time to reach the backward threshold. Thus, the regression rates for regressions of type II should be reduced in cases in which the confidence level of word n is creasing slower (i.e., less frequent and less predictable words).

This prediction, however, cannot be tested by the data of Bicknell and Levy, because they restricted their analysis to regressions targeting word n-1 and in addition excluded regressions that were initiated on the last word in a line. Thus, this hypothesis has to be tested by future research. Also note that in the analysis reported above word n-1 always served as the regression target (in contrast to the assumptions of the IGF model). So, it is hardly to distinguish which properties of word n-1 caused regressions and which qualified them as a potential regression target. This topic also needs more empirical examinations.

#### (2) Regressions to the immediately preceding word

Although the landing positions of regressions are spread over the whole sentence, many studies have shown that the majority of regressive eye movements targets the word immediately preceding the currently fixated word (see e.g., [5, 11, 12] for corresponding evidence). In particular, all current models of eye movement control discussed above (E-Z Reader 10, Model of falling confidence, Glenmore, SWIFT – with some exceptions mentioned above) account only for these instances.

Mitchell et al. [42]; argue (in favor of an automatic regression mechanism) that a regression from word n+1 to word n is the “smallest possible regression” (p. 271). And of course, a regression to word n has some important advantages compared to target words that are farther away from the current fixation: First, the saccade is short and fast, so that less effort for its execution and control is needed. Second, the target word can be processed parafoveally so that the saccade can be guided by using visual input. Third, memory demands are low because the word has been encountered immediately before (see [46] for a detailed discussion of ‘spatial knowledge’ in the context of regressions to word n-1).

In the IGF, however, we argue that regressions to the immediately preceding word can be explained more plausibly by a regression mechanism that is controlled by linguistic factors.

Although they differ in their explanations, both the E-Z Reader and SWIFT model account for the often replicated finding that the processing of word n also affects processing of word n+1 (also known as “lag” or “spillover” effects: 52, 53 see [51] for a discussion). Within the IGF, however, this finding can be explained by the idea that the computation of the confidence level continues after the eyes have moved to word n+1 because the retrieval and integration of linguistic information takes time. Because language processing is organized hierarchically and this hierarchy is assumed to correspond to the time course of sentence interpretation (at least to some degree), the computation of the confidence level of word n on word n+1 is based primarily on higher-order linguistic processing like lexical integration. Thus, an integration failure of word n will often become apparent only on word n+1 (see pattern 2 described above). If this integration fails because the predictions based on the production rules are not met, a regression is triggered. If the production rules reveal that more information about word n is needed (which is assumed to be within the focus of attention, see above) because this information would help to solve the problem, this regression targets word n (see also 18).

Because there are many more instances in which the integration of word n fails due to wrong and/or less specified assumptions about its identity than instances where the integration fails due to wrong / less specified identities of previous words (which is the case for instance in most garden path sentences), the eyes very frequently regress to word n. This explains why the majority of regressions targets the immediately preceding word.

In addition, the backward control mechanism could also have developed a strategy that selects the preceding word. Recall that the strategies applied by the backward control mechanism are assumed to be based on general language knowledge / experience and hence operate on frequency. Thus, in the case the confidence level of none or more than one word (apart from word n-1) did not reach the re-inspection threshold, the backward control mechanism might select the preceding word, because this word often provides the most useful information in order to solve the processing problem.

This view is further supported by the findings of von der Malsburg and Vasishth [12]; indicating that low-capacity readers were less likely to re-read the sentences when faced with garden path sentences. Instead, they used rapid regressions to the word in the pre-disambiguating region more frequently. Since these rapid regressions provide some advantages with regard to memory capacities (as discussed above), this strategy suits readers with low memory capacities.

#### (3) Sentence wrap-up effects

A clear deficit of eye movement models like SWIFT, Glenmore or E-Z Reader is that they attribute regressive eye movements only to processing difficulties (in the case of E-Z Reader) or incomplete word processing / identification (in the case of SWIFT and Glenmore). Whereas this of course covers a wide range of regressions reported in the literature, it excludes some findings at the same time. An important sub-class of regressions, for example, is the increased probability to regress from the end of a sentence (‘sentence wrap-up effect’) which was mentioned earlier.

As discussed above, the IGF is not restricted to processing difficulties, it rather posits that regressions are triggered whenever the predictions made by previous input are not matched. This could either be that the current input conflicts with the predictions (which would lead to a decrease of confidence) or that expected evidence is missing (which would lead to a slower increase of confidence). In the case of regressions from the final region we assume that the latter scenario takes place.

Thus, if the eyes move to the final (or pre-final) word, the confidence level of this word is computed by matching the predictions. But in addition, the punctuation is also received from the visual input (at least parafoveally), which signals a sentence boundary. Sentence boundaries indicate that no additional input for the current sentence interpretation can be received and subsequently no prediction (condition-action pair) can be postponed to later input. Thus, at the end of a sentence an evaluation of the whole sentence interpretation takes place [56, 9, 55];. In the case that this evaluation reveals that more evidence is needed in order to develop a coherent sentence interpretation, a regression is performed to compensate for this information deficit. Of course, the degree of evidence (and of confidence, respectively) in a sentence structure that is assessed to be sufficient (the backward threshold) may depend on factors like task or time pressure.

Since an evaluation of the whole sentence takes place without dealing with a concrete integration problem, it is reasonable to assume that not a single target position based on the production rules can be defined. In contrast, the regression strategy applied selects a target position on the basis of language experience. This prediction fits well with the regression patterns reported by von der Malsburg and Vasishth [11, 12];, which show a clear tendency for readers to regress to the beginning of the sentence and to read the whole sentence again.

#### (4) Gaze durations and regressions

In the beginning we mentioned the counterintuitive finding of Altmann and colleagues [14]; that gaze durations before regressions tend to be shorter relative to gaze durations before progressions. Whereas these results may in general be interpreted in favor of the claim that increased fixation durations and a higher number of regressive eye movements have to be functionally distinguished, the SWIFT model, for example, accounts for this effect by the assumption of saccadic overshoots. In the case of an overshoot, a new saccade program is started immediately. Because it is likely that word n-1 has not been recognized completely and therefore has a high activation level, this word is often targeted by this new saccade.

However, the architecture of the IGF also directly predicts this pattern. Recall that fixation durations are mainly monitored by the forward threshold: As soon as the confidence level of word n reaches the forward threshold, the eyes move to word n+1. If, however, the computation of the confidence level of word n-1 reveals integration difficulties (recall that the computation of the confidence level of word n-1 still continues during a fixation of word n), this causes the confidence level of word n-1 to fall. As a consequence, the fixation of word n is cancelled and a regressive eye movement is performed instead. Because the fixation of word n is cancelled, fixation durations before regressive eye movements tend to be shorter.

But our model makes an additional prediction: Because regressions due to missing evidence are not triggered before the fixation of the current word is completed, we would expect no shorter fixation durations for these types of regressive eye movements (in contrast to regressions due to integration difficulties where a fixation is cancelled and thus the fixation durations are shortened).

#### (5) Regression targets within and outside the perceptual span

The IGF makes a strong prediction with regard to the target selection of regressions: Only words within the perceptual span, which is assumed to comprise about 15–20 characters to the left of the current fixation, can be selected as a regression target by an explicit linguistic computation. Words outside of the perceptual span are assumed to only be selected by a backward strategy. This division should be reflected by the empirical data.

First, it would be quite an unexpected finding if the regression landing sites show, for example, a Gaussian or a linear distribution over the sentence, thus ranging from very short to very long sizes with no further distinctions. We would rather expect that the majority of regressive saccades land within the perceptual span. In addition, we would expect that we are able to find a clear pattern for regressions that land outside the perceptual span because these regression targets are assumed to be selected by a strategy. Murray and Kennedy [41];, for example, identified three different regression strategies in the context of anaphor processing: re-reading ab initio, selective reinspection of some words, or right-to-left backtracking. For the first scenario, for instance, we would expect to see a clear tendency for long regressions to target the beginning of a sentence.

Second, in the case that there exists a well-defined target position from a theoretical linguistic point of view (as for example, in garden path sentences), we would expect that this defined target position is selected as a regression target only if it is within the perceptual span. If the ambiguous word is outside the perceptual span, for instance, no preference for a selection of this word is predicted, unless it is selected by the strategy.

#### (6) Independency of forward and backward threshold

Within the IGF it is assumed that the duration of first-pass reading times is monitored by the forward threshold on one hand and the probability to regress by the backward threshold on the other. Although there is considerable evidence that these two thresholds highly interact (as for example indicated by the speed-accuracy tradeoff), we assume that these two parameters can be set independently.

Thus, we predict that there are cases where a more risky forward strategy does not necessarily lead to an increased probability of regressions. On the other hand, there should be cases where the probability of regressions is increased despite the fact that there are no longer first-pass reading times.

#### (7) Regressions are sensitive to task modulations

Since regressions are assumed to be mediated by both the forward and backward threshold, we would expect that an adjustment of these thresholds should have an impact on the probability of triggering a regression. In particular, top-down influences like task or time pressure should affect the regression behavior during reading leading to more or less regressions, respectively.

### Testing the Information Gathering Framework

In the last section we described the architecture of the IGF and also outlined some predictions that can be derived from the framework. In the following we will look for further empirical evidence by applying these predictions to an experiment conducted by Weiss and colleagues [57];.

In this experiment, 92 English native speakers were asked to read 99 English sentences in total while their eye movements were monitored. These English sentences contained 36 semantic reversal anomalies (SRAs), 39 relative clause sentences (RC) and 24 garden path sentences (GP; see Table 2 for an overview), where each of the RC and GP sentences was followed by a comprehension question.

**Table 2:** Example stimuli used by Weiss et al. (2018). Abbreviations: N = non anomalous, A = anomalous, H = highly associated, L = low associated, SRC = subject relative clause, ORC = object relative clause. The slashes indicate regions for analysis.

**1. Semantic Reversal Anomalies (SRA)**		
(a)	On a sunny afternoon | the girl | is picking | the flower | for the dining table.	NH
(b)	On a sunny afternoon the girl is drawing the flower on a little sketchpad.	NL
(c)	On a sunny afternoon the flower is picking the girl for the dining table.	AH
(d)	On a sunny afternoon the flower is drawing the girl on a little sketchpad.	AL
**2. Relative Clause Sentences (RC)**		
(a)	The chef | that distracted the waiter | sifted the flour onto the counter.	SRC
	(I) Did a chef do something? (II) Did the waiter distract the chef?	easy difficult
(b)	The executives | that the lawyers sued | roused themselves from slumber.	ORC
	(I) Did a policeman do something? (II) Was it the executives who roused themselves?	easy difficult
**3. Garden Path Sentences (GP)**		
	John borrowed | the rake or the shovel | turned out to be sufficient.	
	(I) Is there a shovel? (II) Might the rake have been borrowed?	easy difficult

Crucially, the question difficulty was manipulated between subjects: While one group received only easy comprehension questions (e.g., probing for a word), the other only received questions that required a deeper understanding of the sentence (see [58, 59] for similar manipulations).

The analysis revealed that for anomalous SRA sentences first-pass reading time and go-past time on the verb and object regions was significantly increased which was also shaped by the association between the verb and object. Difficult questions, however, led to significantly longer reading times and more regressions in the sentence-final region, indicated by a significant effect for question difficulty on go-past time and regressions out. But question difficulty had no significant effect on the earlier regions nor interacted with first-pass reading. This was also true for the RC and GP sentences.

To further clarify this pattern, regions 1–4 (SRA sentences) or 1–2 (RC and GP sentences) were merged to one region and the original final region was divided into two regions. The new final region consisted of the last 2–3 words of the sentences. Again, difficult questions induced longer go-past times in the final region for all three sentence types but neither in the first nor in the second region.

Let us now see how the IGF may account for these results.

#### (1) Task manipulation should only affect regression rates

From the perspective of the IGF, we expect that the task manipulation should adjust the backward threshold. Thus, in the easy condition the subjects should have applied a more superficial reading strategy compared to the difficult condition which set the backward threshold to a lower level. More precisely, the IGF makes the strong prediction that this task manipulation should only affect regression rates but not first-pass reading times.

Interestingly, that is exactly the pattern that was found in the data. For the SRAs, the anomaly effect became apparent in first-pass reading irrespective of the task manipulation. However, although the question type did not affect first-pass reading behavior, difficult questions induced significantly more regressions. We may interpret these results as evidence for adjusting the backward threshold independently of the forward threshold by using different reading strategies.

#### (2) Task manipulation should only affect regressions of type II (missing evidence)

A second prediction that can be directly derived from the model’s architecture is that adjusting the backward threshold should only affect regressions of type II (due to missing evidence) but not regressions of type I (due to integration difficulties). Thus, we would expect to find an increasing number of regressive eye movements from the end of a sentence but not from the regions before.

Again, the reported results are in line with this prediction: In all three sentence types there was a significant increase of regressions out of the last 2–3 words of a sentence for the difficult condition. This was not the case for the regions before. Thus, the backward threshold seems to only affect regressions of type II (due to missing evidence) but not regressions of type I (due to integration difficulties).

#### (3) Shorter fixation durations before regressions of type I (integration difficulties)

The IGF makes the strong prediction that fixation durations before regressions should be shorter compared to fixation durations preceding progressions, but only before regressions of type I (due to integration difficulties). This means that we should find shorter fixation durations before regressions in all sentence regions except the last region, where we expect to find either no or a reduced effect of saccade type.

In order to test this prediction, we re-analyzed the data by identifying all inter-word saccades of the SRAs (n=41.800) and categorized them as progressive (n=31.671) or regressive eye movements (n=10.129), respectively. After that we attributed these saccades to the six regions of the sentence (for an example of the regioning-scheme, see Table 2).

**Figure 4:**
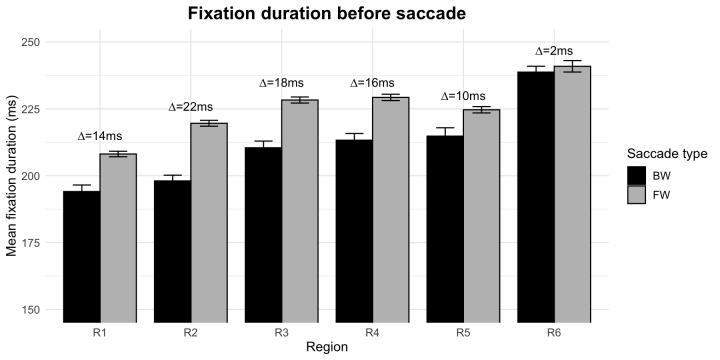
Mean fixation durations before saccades for all inter-word saccades of the SRA sentences, given for each region and saccade type separately. For details of the regioning scheme please refer to Table 1. Abbreviations: R = sentence region, BW = fixation before a regressive saccade, FW = fixation before a progressive saccade, ms = milliseconds.

A first analysis revealed that fixations before regressions were generally shorter (mean 217 ms) than fixations before progressive saccades (mean 222 ms). This difference of about 6 ms was highly significant (*t*(14691) = 4.92, *p*<.001). Looking at the means for the single regions, we also observed that this difference ranged from about 10 to 22 ms in regions 1–5 but dropped to about 2 ms in the last region (see Figure 4). We checked if this difference was significant by fitting a linear mixed effect model of the log fixation duration of the preceding fixation. For this we combined regions 1–5 to a new region (region_early) and compared this with region 6 (region_late), treating SACCADE TYPE and REGION as well as their interactions as fixed effects.

We also used random intercepts for subjects and items and took the maximal random effect structure. Following convention, we treat *t*>|2| as significant.

The results of the linear mixed effect models showed that SACCADE TYPE (*ß* =.07, *SE* =.01, *t* = 6.28) and REGION (*ß* =.10, *SE* =.01, *t* = 7.31) as well as their interaction (*ß* = -.05, *SE* =.02, *t* = -2.48) had a significant impact on fixation durations. Thus, although fixations before regressions were generally shorter (indicated by the significant effect of SACCADE TYPE), this effect was absent in the last region of the sentence (indicated by the significant interaction of SACCADE TYPE X REGION).

This somewhat surprising finding fits well with the prediction made by the IGF: Because only regressions of type I (due to integration difficulties) are triggered in the way that the preceding fixation is cancelled, only fixations before these regressions should be shorter.

Another interesting, although unrelated, finding is that fixation durations generally increase during the course of the sentence (indicated by the significant effect of REGION, see also Figure 4). In terms of the IGF, this points to idea that the amount of information that has to be dealt with increases during the course of the sentence which leads to longer computation times until the forward threshold of confidence is reached. It might be worthwhile to examine the reasons for that in more detail by future research.

#### (4) Regression amplitudes and landing sites of regressive eye movements

**Figure 5:**
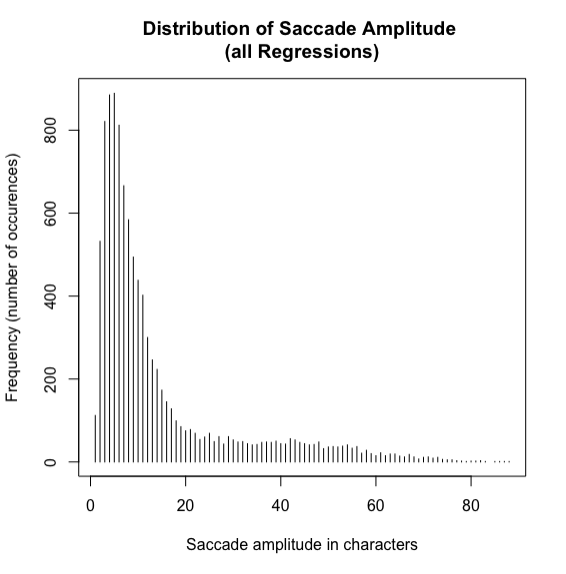
Distribution of saccade amplitude for all regressions in the SRA sentences. X-axis shows the saccade amplitude in characters and the y-axis the number of occurrences.

Although the IGF is not very specific with regard to the landing site distributions yet, we nonetheless would expect that the perceptual span is reflected in the saccade amplitude of regressions. Thus, because regression target selection is assumed to be linguistically constrained but also needs precise spatial knowledge (see Inhoff et al., 2005, for a discussion), the majority of regressions should target a word within the perceptual span. Thus, we first computed the amplitude of all regressive eye movements in the SRA sentences (see Figure 5).

This analysis revealed that 74.81% of all regressions fell within the 15-character window left to the current fixation. However, because we took all regressions, the distance to the beginning of the sentence was reduced for some of them. Thus, we conducted a second analysis and restricted it to regressions that were initiated in the final region only (using the regioning scheme outlined above).

**Figure 6:**
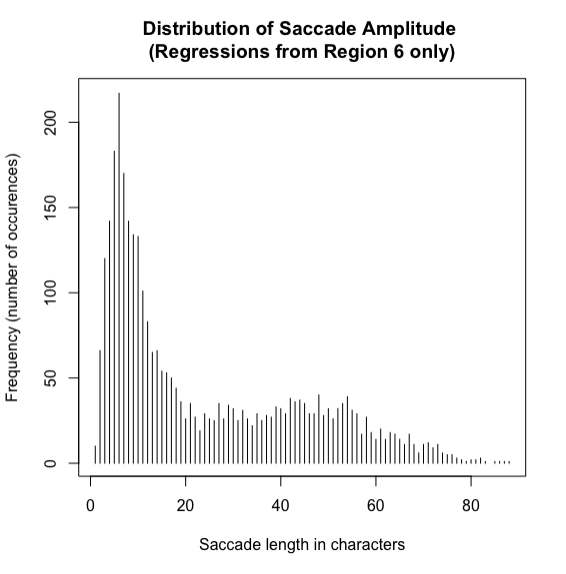
Distribution of saccade amplitude for regressions that were initiated in the final region of the SRA sentences only. X-axis shows the saccade amplitude in characters and the y-axis the number of occurrences.

As becomes apparent from Figure 6, we see a similar pattern, but the proportion of regressions within the 15-character window dropped to 51.61%. Anyway, at about 15–20 characters there seems to be again some kind of invisible boundary for which the probability to be crossed by a regressive eye movement is clearly reduced. This fits well with the assumption of the IGF that the linguistically driven selection of target positions is limited by the perceptual span. From this data we may conclude that the perceptual span comprises about 15–20 characters to the left of the current fixation for regressive eye movements, although certainly more research is needed here.

Because the number of characters varied within sentences and regions, the saccade amplitude it not very meaningful with regard to the actual location in the sentence where the regressions landed. Thus, we further investigated the landing site distributions by aligning the target positions with the six sentence regions defined above.

**Figure 7:**
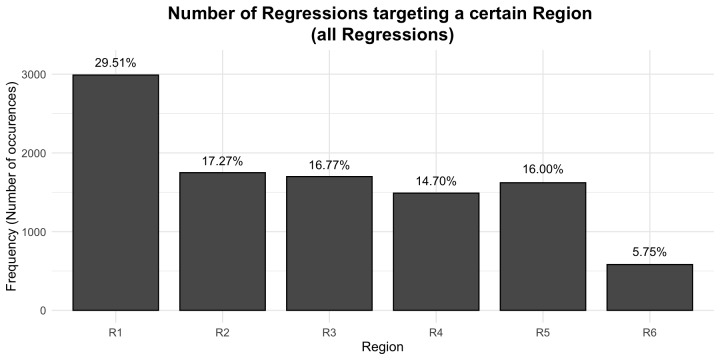
Number of all regressions of the SRA sentences targeting a certain sentence region (for details of the sentence regioning scheme, please refer to Table 1). The percent values represent the proportion of all regressions.

When taking all regressions into account we see a clear tendency to target the first region of the sentence (29.51%), thus probably resulting in subjects re-reading the whole sentence again (see Figure 7). When only focusing on regressions from the final region, we see again an increased tendency to regress from the sentence beginning (14.45%) but substantially more regressions (33.18%) landed in the pre-final region (which is a quite expected pattern given the results of the amplitude analysis above). These results are fully in line with the predictions of the IGF: The majority of regressions target a position within the perceptual span but if they cross this span, most likely a strategy is applied which is for subjects to re-read the whole sentence again. This also fits well with the regression patterns reported by [11, 12].

However, because the experiment was not designed to conduct an analysis on the landing-site distributions, factors like region length were not controlled. Thus, these results just give a first impression but stress the need to investigate the target pattern of regressive eye movements in more detail by future research.

## Conclusions

In this article we introduced a new eye movement control framework that especially focuses on regressive eye movements during reading: The Information Gathering Framework (IGF). Based on the FC model proposed by Bicknell and Levy, the basic idea of the IGF is that a confidence level for each word is computed while being monitored by two independent thresholds: the forward and the backward threshold, respectively. These two thresholds shape the eye movement behavior by increasing fixation durations or triggering a regression. In addition, a third threshold, the re-inspection threshold, monitors the regression target selection. In this way, the IGF does not only account for regressive eye movements but also provides a framework that is able to model eye movement control during reading across different scenarios.

Importantly, within the IGF it is assumed that two different types of regressive eye movement exist which differ with regard to their releases (integration difficulties vs. missing evidence) but also with regard to their time course. By re-analyzing an experiment of Weiss et al. [57]; we found, inter alia, clear evidence for shorter fixation durations before regressive saccades relative to progressive saccades, with the exception of the last region. These results confirm the predictions of the IGF. The IGF also proposes that a linguistically driven computation of the target positions should only be possible within the perceptual span. Our data suggests that a 15–20-character window to the left of the current fixation indeed plays an important role within the target selection process. We conclude that this area of about 15–20 characters is likely to cover the size of the perceptual span during a regressive eye movement.

However, both the architecture and the testing of the IGF are not fully sufficient yet but only provide a first tool for future research. So, it became clear that regressive eye movements are not just an ‘error message’ but seem to play an important role in developing a successful and fast reading strategy. Nonetheless, the details of their role for word identification, but also for sentence and text reading as well as their interaction with language comprehension are still unclear (but see e.g. [30] for a discussion of this problem). However, the current framework may provide a promising new perspective on comprehension monitoring in the way that it explicitly covers high-level linguistic processing and its interaction with re-reading of words all of over the sentence (and not only re-reading of the immediately preceding word).

Further topics that still need more empirical examination are the time-course and landing-site distributions of regressive eye movements, especially the perceptual span and target selection. But we are convinced that the IGF allows us to derive precise questions for future research which will in turn give us good answers to understand the role of regressive eye movements during reading in more detail.

## Ethics and Conflict of Interest

The author declares that the contents of the article are in agreement with the ethics described in http://biblio.unibe.ch/portale/elibrary/BOP/jemr/ethics.html and that there is no conflict of interest regarding the publication of this paper. The model presented in this paper is part of the doctoral dissertation of AFW, submitted at Philipps-University of Marburg and online available at http://archiv.ub.uni-marburg.de/diss/z2017/0719
